# Hepatitis Awareness Month and National Hepatitis Testing Day — May 2014

**Published:** 2014-05-09

In the United States, May is Hepatitis Awareness Month, and May 19 is National Hepatitis Testing Day. Although care and treatment can be life-saving, many of the estimated 800,000 to 1.4 million persons living with hepatitis B virus (HBV) infection and the estimated 3 million persons living with hepatitis C virus (HCV) infection are unaware of their infection and are not receiving necessary care and treatment (1). Guided by the goals of the 2014 U.S. Department of Health and Human Services *Action Plan for the Prevention, Care, and Treatment of Viral Hepatitis* (1), CDC is working to expand access to HBV and HCV testing, care, and treatment. This issue of *MMWR* reports on the progress of these CDC activities in reaching the national prevention goals.

The first report examines projects (based on the Project ECHO model of videoconference and case-based learning) to strengthen HCV primary care capacity in Arizona and Utah. In the second report, programs in three sites (New York City, Minneapolis-St. Paul, and San Diego) targeted HBV testing for persons born in countries where HBV infection is endemic (≥2% prevalence). Both programs were successful in reaching persons in underserved populations (in predominantly rural settings for hepatitis C and among foreign-born persons for hepatitis B) and linking them to appropriate care and treatment. Broader expansion of programs like these will help prevent HBV and HCV transmission and disease.
